# Salivary HOTAIR and PVT1 as novel biomarkers for early pancreatic cancer

**DOI:** 10.18632/oncotarget.8323

**Published:** 2016-03-23

**Authors:** Zijun Xie, Xiaoliang Chen, Jianzhong Li, Yunwei Guo, Haijiao Li, Xuemei Pan, Jie Jiang, Huiling Liu, Bin Wu

**Affiliations:** ^1^ Department of Gastroenterology, The Third Affiliated Hospital, Sun Yat-Sen University, Guangzhou, China

**Keywords:** HOTAIR, PVT1, pancreatic cancer, biomarker, diagnosis

## Abstract

Sensitive and non-invasive biomarkers for pancreatic cancer (PC) are lacking. We aimed to identify salivary long non-coding RNAs (lncRNAs) as biomarkers in diagnosis of resectable PC. Five well-documented lncRNAs: H19, HOTAIR, HOTTIP, MALAT1, PVT1, which are most closely associated with pancreatic cancer from previous studies were selected as putative lncRNA biomarkers. Their expression in pancreatic tissues and saliva of cancer patients and healthy controls was measured by quantification polymerase chain reaction (qPCR). Compared with benign pancreatic tumour (BPT) and normal pancreatic tissues (NPT), HOTAIR, HOTTIP and PVT1 were significantly up-regulated in pancreatic cancer tissues (PCT). As compared to BPT or healthy groups, the salivary levels of HOTAIR and PVT1 were significantly higher in PC group. They were significantly reduced after the curative pancreatectomy. Both salivary lncRNAs distinguished PC patients from healthy controls and BPT patients with sensitivities and specificities ranging from 60–97%. The expression of salivary HOTAIR and PVT1 did not differ significantly between healthy controls and any one of eight leading cancers worldwide. Collectively, our findings indicate that salivary HOTAIR and PVT1 show potential as novel non-invasive biomarkers for detecting PC.

## INTRODUCTION

Pancreatic cancer (PC) has the poorest survival rate among all human cancer types. Its mortality is almost identical to incidence. The median survival time of PC is only 4 months [1]. Although, the 5-year survival of post-operation for PC is only 20%, surgical resection is the only potentially curative treatment. However, only 10–20% of patients are diagnosed with the disease at a resectable stage [2].

The pancreas is a small organ that is located deep within the body, which is part of the problem in detection via imaging modalities. More importantly, there are no specific symptoms for PC, and sensitive biomarkers are lacking. Hence, PC is often diagnosed at late stage. To date, carbohydrate antigen 19–9 (CA19-9) is the only biomarker related to PC for which U.S. Food and Drug Administration (FDA)-cleared diagnostics exist [3]. But serum CA19-9 has no role in screening asymptomatic populations, and shows a sensitivity and specificity of 79–81% and 82–90% respectively for the diagnosis of PC in symptomatic patients. Vital limitations to CA19-9 diagnosis in PC include poor sensitivity, false negative results in Lewis negative phenotypes of blood groups (5–10%) and increased false positivity in patients with obstructive jaundice (10–60%) [4]. Thus, there is an urgent need to find a sensitive and noninvasive biomarker for screening early pancreatic cancer.

Long non-coding RNAs (lncRNAs) are transcripts longer than 200 nucleotides. lncRNAs activate several biological processes such as transcription, translation, gene expression etc. LncRNAs show great tissue specificity, making them attractive in the search of novel cancer biomarkers in body fluid samples. According to previous reports, lncRNAs: H19, HOTAIR (HOX transcript antisense intergenic RNA), HOTTIP (HOXA distal transcript antisense RNA), MALAT1 (metastasis-associated lung adenocarcinoma transcript 1), PVT1 (plasmacytoma variant translocation 1) are most closely associated with PC. There are all up-regulated in PC tissues or cell lines, exhibit pro-oncogenic activities and correlate with unfavorable prognosis. H19 promotes PC cell invasion and migration partially by enhancing HMGA2-mediated epithelial-mesenchymal transition (EMT) via antagonizing let-7 [5]. Gene set enrichment analysis indicates that HOTAIR stimulates gene sets mainly associated with cell proliferation and cell cycle progression. HOTAIR knockdown in PC cells decreases cell proliferation, alters cell cycle progression, induces apoptosis and inhibits tumour growth in mouse xenograft model [6]. HOTTIP enhances cell proliferation, invasion, and chemoresistance by activating HOXA13 [7]. MALAT1 knowdown inhibits PC cell proliferation and reduces cell migration and invasion *in vitro*. The underlying mechanisms are possibly associated with inducing G2/M cell cycle arrest, promoting cell apoptosis, suppressing EMT and decreasing cancer stem-like properties [8]. Functional inactivation of the PVT1 contributes to enhanced Gemcitabine sensitivity in human pancreatic cancer ASPC-1 cells, vice versa [9].

Very limited studies report the diagnostic performance of circulating lncRNAs for detecting PC. Plasma fragments of HOTTIP-005 and RP11-567G11.1 were significantly up-regulated in the PC patients. The sensitivity was 89% and 75.6% and specificity was 68.3% and 66.7%, respectively, for discriminating PC from non-PC [10]. But blood collection is still invasive, and can cause pain and infection. A large blood supply flows into the salivary glands. Most compounds found in blood are also present in saliva, such as DNA, RNA, protein, drugs, and viruses. In the USA, commercial products for saliva-based diagnosis of drug abuse and HIV infection and for assessing hormone levels and various toxicology issues have been approved by the FDA and are in widespread use [11]. Studies report that salivary transcripts, proteins, metabolites, and other molecules may serve as biomarkers to detect oral cancer, breast cancer, lung cancer, ovarian cancer, esophageal cancer, Sjögren's syndrome, and other oral and systemic diseases [12, 13]. Thus, saliva can be a desirable body fluid for biomarker detection in clinical applications. The functions of H19, HOTAIR, HOTTIP, MALAT1, and PVT1 in PC have been partly understood, but their salivary levels for detecting PC remained unclear. We aimed to explore whether they could serve as non-invasive biomarkers in saliva to detect resectable PC in this study.

## RESULTS

### Basic participant information

From 2011 to 2015, 55 patients with resectable pancreatic cancer, 20 patients with benign pancreatic tumour were enrolled in this study. Their diagnosis was confirmed by pathology. 55 healthy individuals matched with PC patients in age, gender, and ethnicity were recuited. Their basic information is summarized in Table 1.

**Table 1:** Demographic and clinical information of enrolled participants

Demographic variables	Characteristics	Pancreatic cancer (*n* = 55)	Healthy control (*n* = 55)	Benign pancreatic tumour (*n* = 20)	*P* Value^*^
Age (years)					0.141
	Mean ± SD	60.7 ± 12.5	57.1 ± 11.3	51.3 ± 13.2	
	Median (range)	61(13–81)	57 (23–71)	49.0 (34–79)	
Sex					0.842
	Male	36	35	14	
	Female	19	20	6	
Ethnicity	Han Chinese	55	55	20	1
CA19-9					
	≥ 37 U/mL	25	0	10	
	< 37 U/mL	30	55	10	
Tumour position					
	Head and neck	42		11	
	Body and tail	13		9	
Tumour size					
	≥ 4 cm	30		6	
	< 4 cm	25		14	
Cancer staging					
	I	9			
	II	14			
	III	32			
Histopathological type					
	Ductal adenocarcinoma	49	Chronic inflammation	10	
	Acinar cell carcinoma	3	Papillary epithelioma	4	
	Mucinous cystadenocarcinoma	3	#Others	6	

* The *P*-value represents the difference between pancreatic cancer patients and healthy controls. #Other histopathological types include autoimmune pancreatitis (*n* = 2), mucinus cystadenoma (*n* = 1), fibroplasias (*n* = 1), leiomyoma (*n* = 1), granulomatous inflammation (*n* = 1).

### The expression of the putative lncRNA biomarkers in tissue and saliva

**Figure 1:**
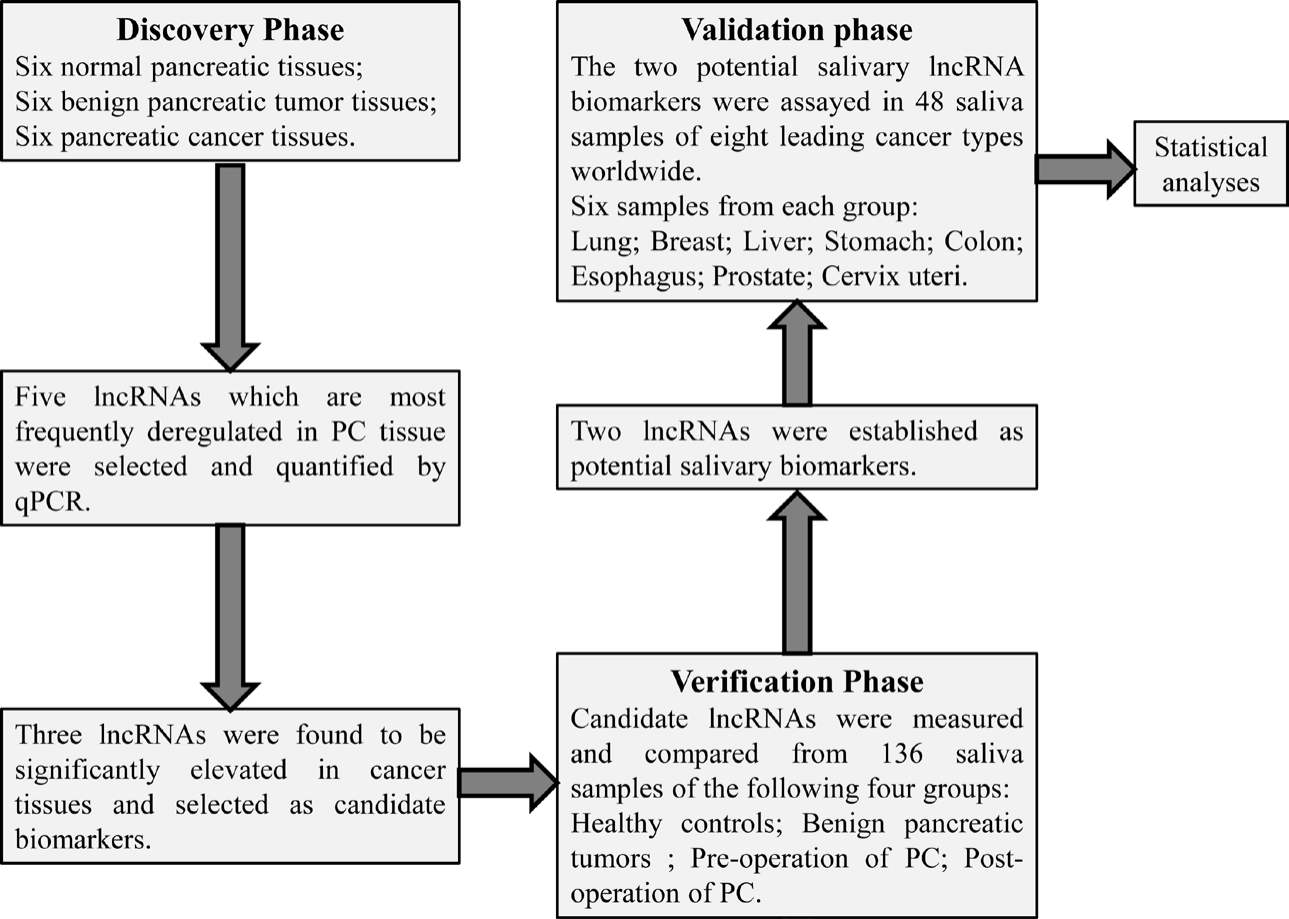
The flowchart of this study Five well-documented lncRNAs (H19, HOTAIR, HOTTIP, MALAT1, PVT1) closely associated with pancreatic cancer from previous studies were selected as putative lncRNA biomarkers. Their expression levels were firstly quantified and compared within different types of pancreatic tissue in the discovery phase. In the verification phase, their expression in saliva was compared within healthy controls, benign pancreatic tumour, pre-operative samples of pancreatic cancer (PC), and post-operative samples of PC. HOTAIR and PVT1 were established as potential salivary lncRNA biomarkers, and their expressions were also analyzed in saliva of patients with any one of eight leading cancer types worldwide. Finally the discriminatory power of salivary HOTAIR and PVT1 was obtained by statistical analyses.

This study consisted of a discovery phase and a verification phase, followed by a validation phase. Five well-documented lncRNAs (H19, HOTAIR, HOTTIP, MALAT1, PVT1) most closely associated with PC from previous studies were selected as putative lncRNA biomarkers. Their expression levels of different kinds of pancreatic tissues were analyzed in the discovery phase to select candidate biomarkers. Potential biomarkers were established in the verification phase based on their differential salivary levels between the healthy controls and the patients. In validation phase, the specificities of the potential salivary biomarkers were further validated by comparing their expression between healthy controls and patients with any one of eight leading cancers worldwide (Figure 1).

**Figure 2:**
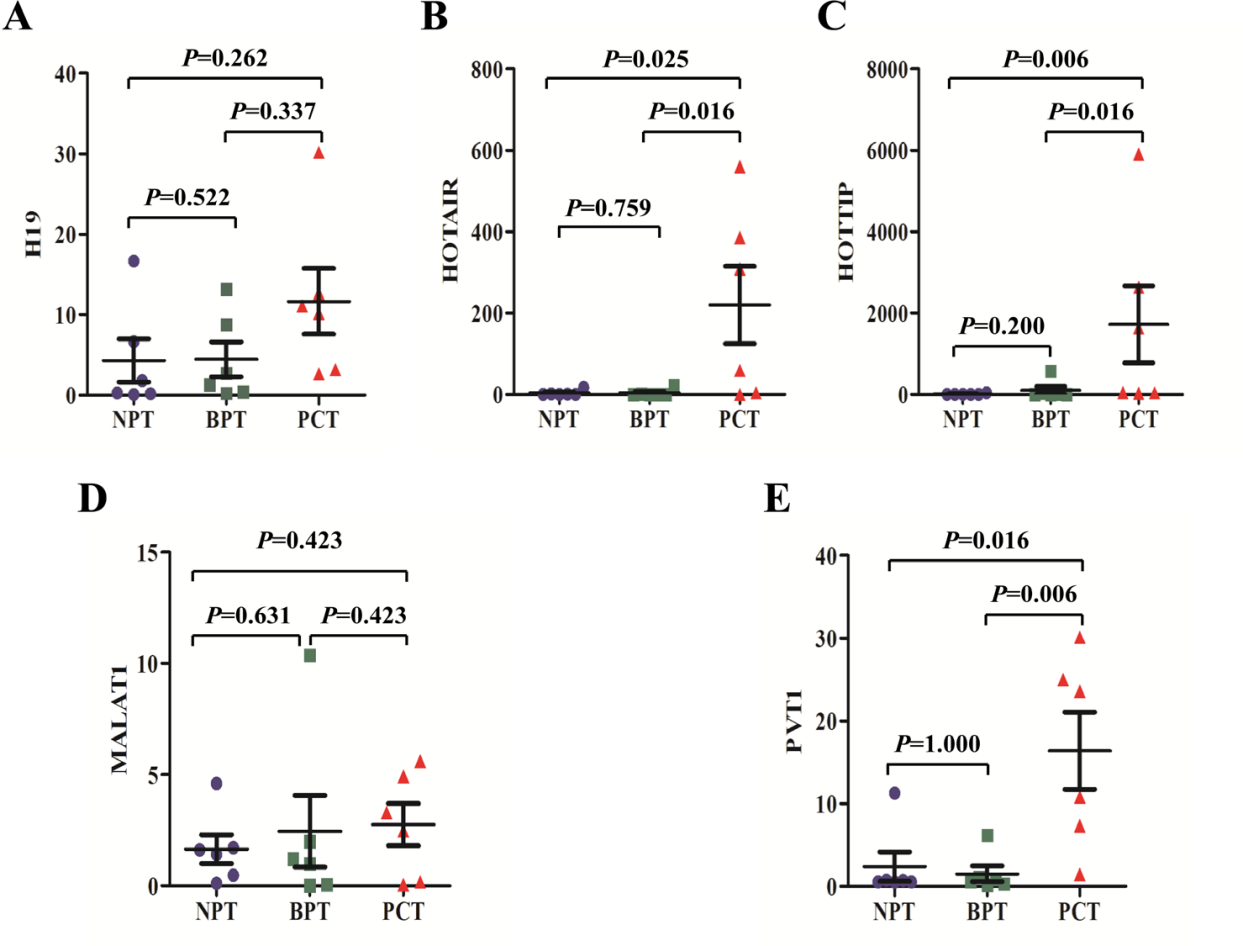
The expression of putative lncRNA biomarkers in pancreatic tissues The expression of H19 (**A**) and MALAT1 (**D**) did not differ significantly between pancreatic cancer tissues (PCT) and normal pancreatic tissues (NPT) or benign pancreatic tumour (BPT) tissues. Meanwhile, as compared to NPT or BPT tissues, the expression of HOTAIR (**B**) HOTTIP (**C**) and PVT1 (**E**) exhibited significant elevation in PCT. There were no statistical differences in H19, HOTAIR, HOTTIP, MALAT1 and PVT1 levels between NPT and BPT tissues. The bars represent the standard errors, and the lines across the bars denote the means.

Compared with normal pancreatic tissues (NPT), the expression of HOTAIR, HOTTIP, and PVT1 exhibited significant elevation in pancreatic cancer tissues (PCT) (*p* values were 0.025, 0.006, and 0.016, respectively). Meanwhile, H19 and MALAT1 levels did not significantly differ between the two groups (*p* values were 0.262 and 0.423, respectively). And H19, HOTAIR, HOTTIP, MALAT1, and PVT1 levels did not significantly differ between NPT and benign pancreatic tumours (BPT) tissues (*p* values were 0.522, 0.759, 0.200, 0.631, and 1.000, respectively). Compared with BPT, HOTAIR, HOTTIP, and PVT1 were also showed significantly higher expression levels in PCT (*p* values were 0.016, 0.016, and 0.006, respectively). But H19 and MALAT1 showed no significant differences between the two groups, (*p* values were 0.337 and 0.423, respectively) (Figure 2).

**Figure 3:**
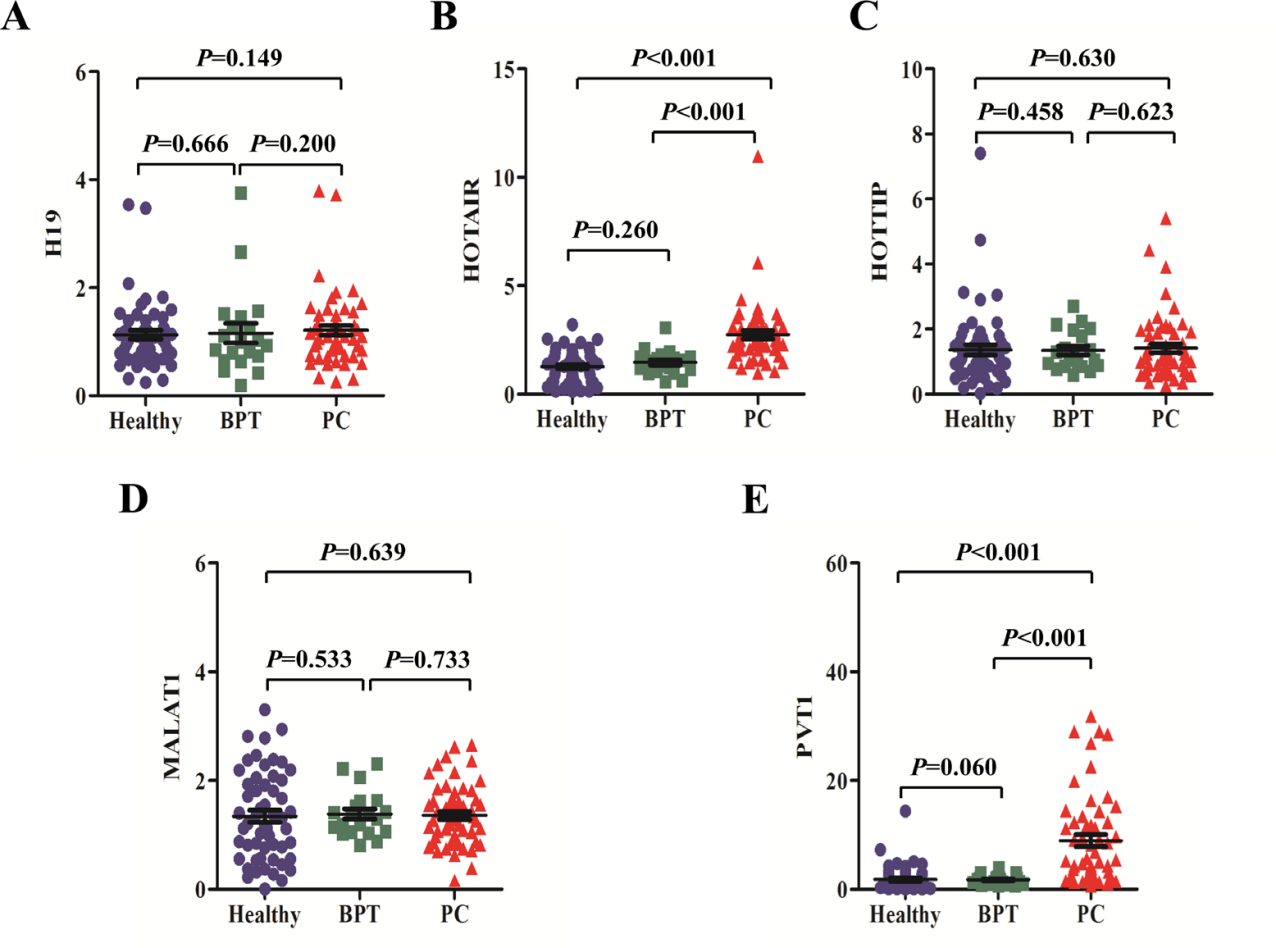
The expression of putative lncRNA biomarkers in saliva There were no expression differences of salivary H19 (**A**) HOTTIP (**C**) and MALAT1 (**D**) between healthy, BPT, and PC groups. But salivary HOTAIR (**B**) and PVT1 (**E**) levels showed significant over-expression in PC group compared with healthy or BPT group. No significant difference was observed about salivary H19, HOTAIR, HOTTIP, MALAT1 and PVT1 levels between healthy and BPT groups.

Compared with healthy controls, salivary HOTAIR and PVT1 showed significant over-expression in PC patients (both *p* values were < 0.001), but there were no significant differences of salivary H19, HOTTIP, and MALAT1 levels between the two groups (*p* values were 0.149, 0.630 and 0.639, respectively). Salivary levels of H19, HOTAIR, HOTTIP, MALAT1 and PVT1 did not differ significantly between healthy and BPT groups (*p* values were 0.666, 0.260, 0.458, 0.533 and 0.060, respectively). Additionally, significant differences were showed between salivary levels of HOTAIR and PVT1 in PC and BPT patients (both *p* values were < 0.001), but no significant differences were obtained in salivary H19, HOTTIP, and MALAT1 levels between the two groups (*p* values were 0.200, 0.623, 0.733, respectively) (Figure 3).

### The expression alteration of salivary HOTAIR and PVT1 after the curative pancreatectomy

**Figure 4:**
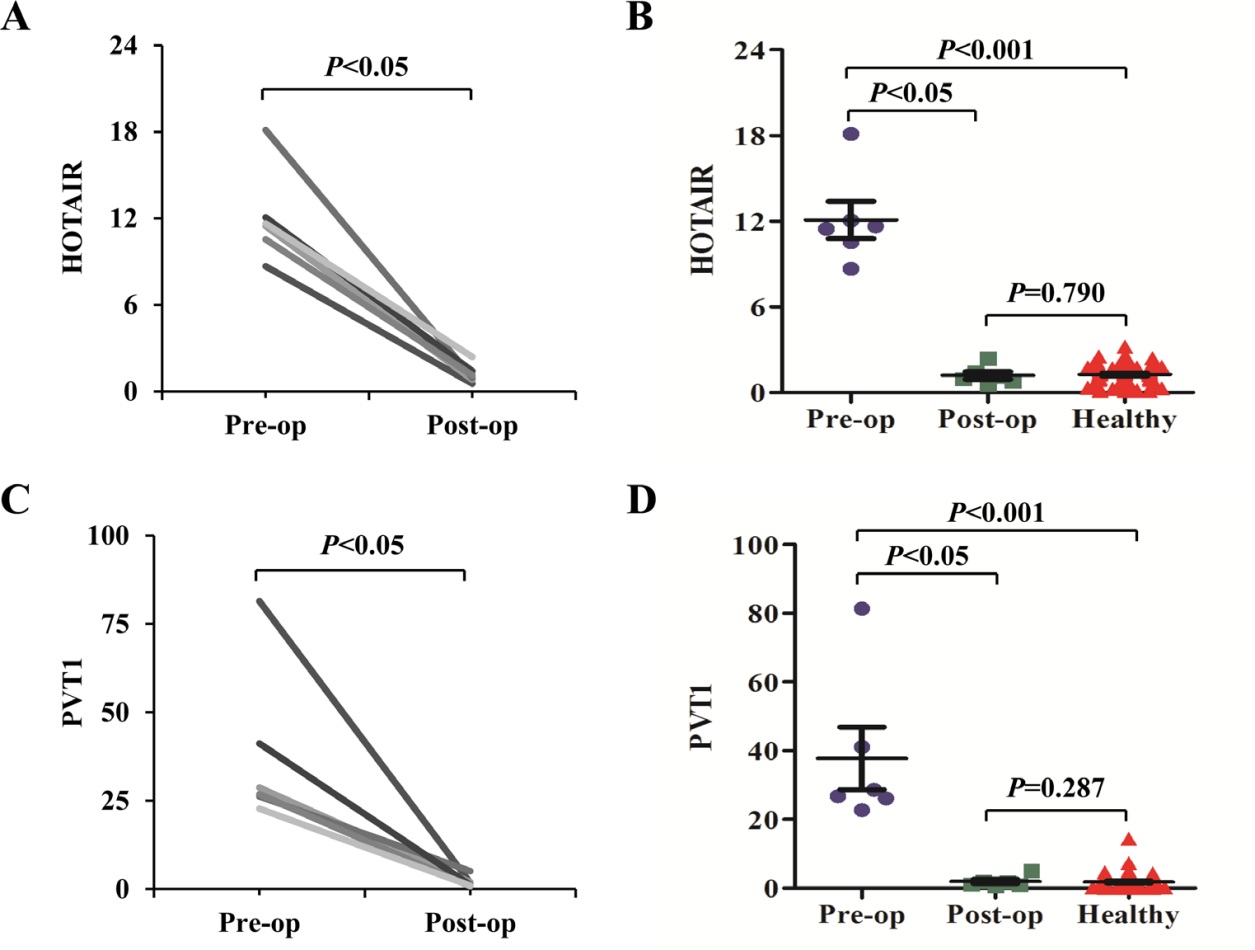
The expression alteration of salivary HOTAIR and PVT1 after the curative pancreatectomy Salivary HOTAIR (**A**, **B**) and PVT1 (**C**, **D**) levels were significantly reduced in the post-operative (Post-op) samples when compared to the pre-operative (Pre-op) samples. No significant differences were obtained when their pre-operative levels were compared with the controls.

In the randomly selected six PC patients receiving curative pancreatectomy, their salivary levels of HOTAIR and PVT1 were compared before and after the surgery. The six patients were followed up at least six months, and CT or/and MRI did not indicate cancer recurrence in them. The post-operative saliva samples of the patients were collected over six months after the surgery. Salivary HOTAIR and PVT1 levels were significantly reduced after surgery (both *p* values were < 0.05) (Figure 4). And their post-operative levels also returned to normal levels judging from no significant differences of their levels between the post-operative and healthy groups (*P* values were 0.790 and 0.287, respectively).

### The discriminatory power of salivary HOTAIR and PVT1 to detect PC

Receiver Operating Characteristic (ROC) curves were constructed and used to evaluate the discriminatory power of salivary HOTAIR and PVT1 for differentiating between two groups. An optimal cut-off value is needed for ROC curve to define the discriminatory power. When the Youden index (Youden index = sensitivity + specificity −1) reaches the maximum value, the corresponding cut-off value will yield the highest sum of sensitivity and specificity.

**Figure 5:**
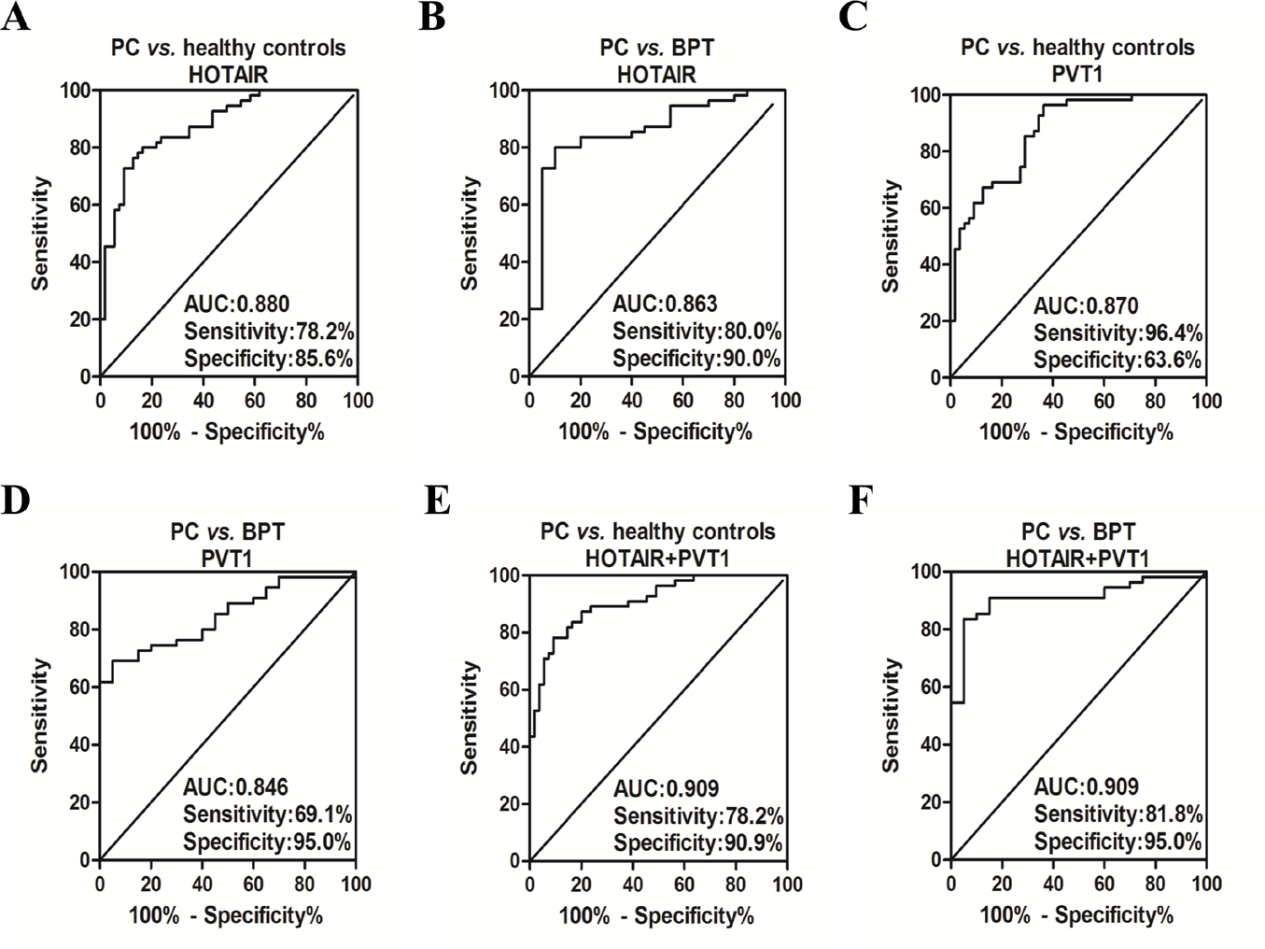
The ROC curves of salivary HOTAIR and PVT1 for detecting PC Salivary HOTAIR differentiated PC from healthy control with sensitivity of 78.2%, and specificity of 85.6% (**A**). And it differentiated PC from BPT with sensitivity of 80.0%, and specificity of 90.0% (**B**). Salivary PVT1 yielded sensitivity of 96.4%, and specificity of 63.6% for distinguishing PC from healthy controls (**C**). And it yielded sensitivity of 69.1%, and specificity of 95.0% for distinguishing PC from BPT (**D**). The sensitivity of salivary HOTAIR + PVT1 combination was 78.2%, and specificity was 90.9% for discriminating PC from healthy controls (**E**). And the combination discriminated PC from BPT with sensitivity of 81.8%, and specificity of 95.0% (**F**).

After calculation of the Youden index, if the cut-off value was determined to be 2.168921, salivary HOTAIR differentiated PC from healthy control with sensitivity of 78.2%, and specificity of 85.6% (Figure 5A). And it differentiated PC from BPT with sensitivity of 80.0%, and specificity of 90.0% (Cut-off value = 1.98554) (Figure 5B). Additionally, salivary PVT1 yielded sensitivity of 96.4%, and specificity of 63.6% for distinguishing PC from healthy controls (Cut-off value = 1.23991) (Figure 5C). And it yielded sensitivity of 69.1%, and specificity of 95.0% for distinguishing PC from BPT (Cut-off value = 3.11795) (Figure 5D). Through logistic regression model, both of the discriminatory power of salivary HOTAIR and PVT1 were combined. Statistically, the area under the curve (AUC) is bigger, the discriminatory power is better. Thus, judging by the AUCs, the discriminatory power of the combination was improved. According to the logistic regression model, and supposing that the salivary levels of HOTAIR and PVT1 are X1 and X2, the combination formula was F1 = −4.183 + 1.716X1 + 0.265X2. Accordingly salivary HOTAIR + PVT1 combination differentiated PC from healthy control with sensitivity of 78.2%, and specificity of 90.9% (Cut-off value = 0.17256) (Figure 5E). And it differentiated PC from BPT with sensitivity of 81.8%, and specificity of 95.0% (The combination formula F2 = −3.917 + 1.66X1 + 0.59X2, and cut-off value = 0.770875) (Figure 5F). The results are summarized in Table 2.

**Table 2:** The discriminatory power of salivary HOTAIR and PVT1 for the detection of resectable pancreatic cancer

		PC *vs.* healthy control		PC *vs.* BPT				
**LncRNA**	***P***	**AUC**	**Sensitivity**	**Specificity**	***P***	**AUC**	**Sensitivity**	**Specificity**
HOTAIR	< 0.001	0.880	78.2%,	85.6%	< 0.001	0.863	80.0%	90.0%
PVT1	< 0.001	0.870	96.4%	63.6%	< 0.001	0.846	69.1%	95.0%
HOTAIR + PVT1	< 0.001	0.909	78.2%	90.9%	< 0.001	0.909	81.8%	95.0%

### The discriminatory power of salivary HOTAIR and PVT1 for detecting PC patients with CA19-9 < 37 U/ml

**Figure 6:**
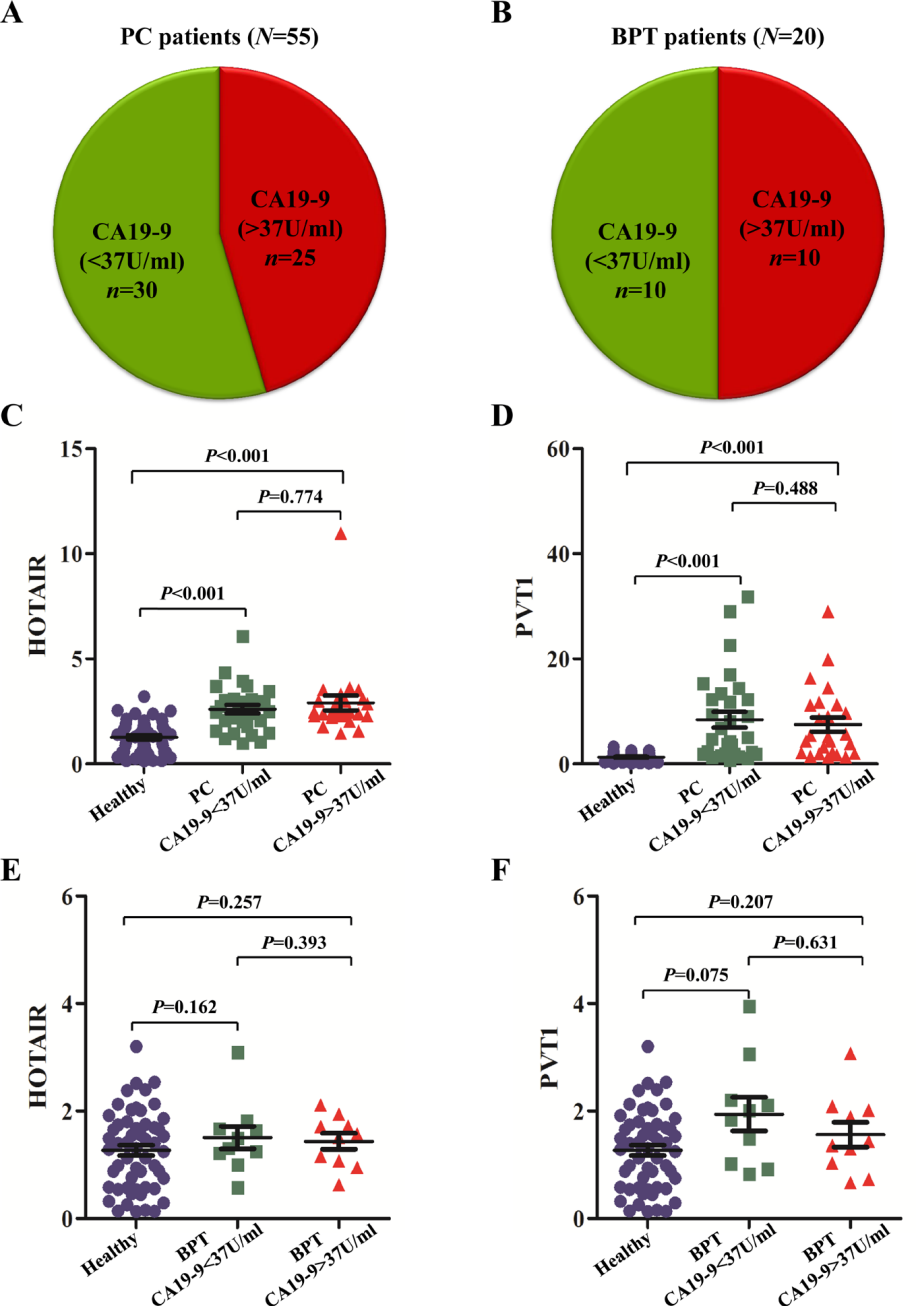
The aberrant expression of salivary HOTAIR and PVT1 in PC with different CA19-9 ranges . In this study, 25 of 55 (45.5%) PC patients showed CA19-9 levels exceeding the normal limit of < 37 U/ml (**A**). Meanwhile, 10 of 20 (50%) BPT patients exhibited CA19-9 > 37 U/ml (**B**). Compared with healthy controls, the expression of salivary HOTAIR (**C**) and PVT1 (**D**) in both groups of PC with serum CA19-9 < 37 U/ml and > 37 U/ml showed significant up-regulation, but their expression levels were not significantly deregulated in both groups of BPT with serum CA19-9 < 37 U/ml and > 37 U/ml (**E**, **F**).

To date, CA19-9 is the only biomarker related to PC for which FDA-cleared diagnostics exist. We analyzed whether the potential lncRNA biomarkers in saliva could show better discriminatory power in detecting PC than that of serum CA19-9. According to the international criteria, the upper limit of serum CA19-9 for healthy people is 37 U/ml. In this study, 25 of 55 PC patients showed CA19-9 levels exceeding this limit, indicating that serum CA19-9 showed a sensitivity of 45.5% (25/55) for the detection of resectable PC (Figure 6A). Meanwhile, 10 of 20 BPT patients exhibited CA19-9 levels > 37 U/ml, implying that serum CA19-9 showed a specificity of 50% (10/20) (Figure 6B) for discriminating patients with benign pancreatic tumours from malignant ones. Compared with healthy controls, the expression of salivary HOTAIR and PVT1 in both groups of PC with serum CA19-9 < 37 U/ml and CA19-9 > 37 U/ml showed significant up-regulation (all *P* values were < 0.001) (Figure 6C, 6D). But their expression levels were not significantly higher in both groups of BPT with serum CA19-9 < 37 U/ml and CA19-9 > 37 U/ml (Figure 6E, 6F).

**Figure 7:**
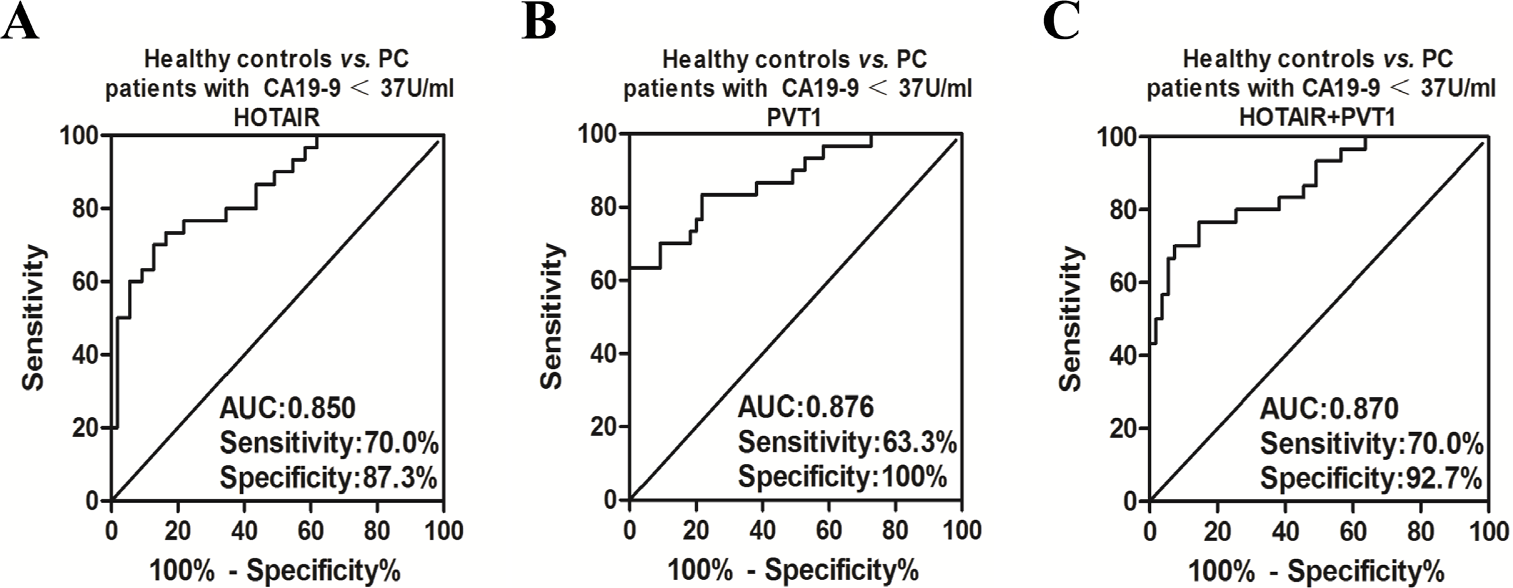
The ROC curves of salivary HOTAIR and PVT1 for detecting PC with different CA19-9 ranges Salivary HOTAIR discriminated PC with serum CA19-9 < 37 U/ml from healthy controls with sensitivity and specificity of 70.0% and 87.3%, respectively (**A**). And salivary PVT1 showed sensitivity and specificity of 63.3% and 100% in discriminating PC with serum CA19-9 < 37 U/ml from healthy controls (**B**). After combining their discriminatory power, their combined sensitivity and specificity for detecting PC with serum CA19-9 < 37 U/ml were 70.0%, 92.7%, respectively (**C**).

According to the ROC curve analysis, salivary HOTAIR discriminated PC with serum CA19-9 < 37 U/ml from healthy controls with sensitivity and specificity of 70.0% and 87.3%, respectively (Cut-off value = 2.063118) (Figure 7A). And salivary PVT1 discriminated PC with serum CA19-9 < 37 U/ml with sensitivity and specificity of 63.3% and 100%, respectively (Cut-off value = 3.473288) (Figure 7B). After combining their discriminatory power by the logistic regression model, their combined sensitivity and specificity for detecting PC with serum CA19-9 < 37 U/ml were 70.0%, 92.7%, respectively (The combination formula F3 = −3.965 + 0.627X1 + 0.937X2, and cut-off value = −0.13145) (Figure 7C).

### The expression of the salivary HOTAIR and PVT1 in other leading cancers

**Figure 8:**
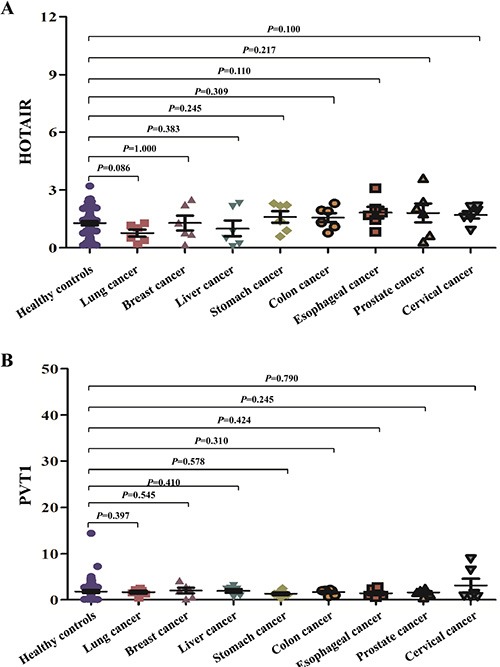
The expression of the salivary HOTAIR and PVT1 in other leading cancers The expression of salivary HOTAIR (**A**) and PVT1 (**B**) did not differ significantly between healthy controls and any one of eight leading cancers worldwide.

In order to preliminarily evaluate the specificities of the two potential lncRNA biomarkers, their expression levels were assessed in total 48 saliva samples of eight leading causes of cancer-related deaths worldwide, including lung, breast, liver, stomach, colon, esophagus, prostate, cervix uteri cancers (six samples from each cancer) [14], and compared with that of healthy controls. The expression of salivary HOTAIR and PVT1 did not differ significantly between any one of the aforementioned cancers and healthy controls (Figure 8).

## DISCUSSION

To our knowledge, this is the first study to report salivary lncRNA biomarker for detecting human disease. According to the measurement by qPCR and statistical analyses, salivary HOTAIR and PVT1 were potentially useful biomarkers, with good discriminatory power for differentiating PC from healthy controls and BPT. The sensitivities and specificities ranged from 60–100%. After the curative pancreatectomy without recurrence for over six months, the expression levels of salivary HOTAIR and PVT1 in the PC patients were significantly reduced and return to normal levels. This result primarily implied that the aberrant expression of HOTAIR and PVT1 was closely associated with or mainly derived from distal pancreatic tumour. Additionally, this pilot study preliminarily found that the levels of salivary HOTAIR and PVT1 in patients with any one of the eight leading cancers worldwide did not differ significantly with that of healthy controls. And the PC patients in this study were recruited based on no concurrent oral, systemic and infectious diseases. Thus, although the sample sizes of other leading cancers were small, this result implies that salivary HOTAIR and PVT1 show potential to be a novel and specific biomarker for PC. But studies with larger sample sizes and other less common cancers are still needed to prove their specificities to PC. Finally, the salivary levels of the two lncRNAs did not differ significantly between BPT and healthy groups, indicating that the two lncRNAs could be deregulated in saliva only when PC developed.

The most common serum biomarker used for PC is CA19-9, which is over-expressed in pancreatic and other gastrointestinal tumours, including esophageal and gastric cancers. Additionally, it cannot distinguish between cancer and chronic pancreatitis and possibly other disease states with chronic inflammation [15]. In this study, less than one half of PC patients and one half of BPT patients showed elevated levels of serum CA19-9. Salivary HOTAIR and PVT1 were not significantly elevated in BPT patients and patients with any one of eight leading cancers worldwide. Hence, in terms of the non-invasiveness and convenience, and better diagnostic performance, salivary HOTAIR and PVT1 might take the place of CA19-9 for PC detection in the future. But these findings still need large-scale validation. Statistically, when the discriminatory power of each biomarker was combined via logistic regression model, the combined discriminatory power would be improved judging by the AUCs. Interestingly, when the discriminatory power of salivary HOTAIR and PVT1 for detecting PC with serum CA19-9 < 37 U/ml was combined, the combined AUC did not increase. The reason might be that both salivary levels of HOTAIR and PVT1 were normal in some PC patients with serum CA19-9 < 37 U/ml. Thus, both makers cannot make up for each other to detect PC with serum CA19-9 < 37 U/ml. Although mRNAs have been considered to be fragile and easily degrade, lncRNAs were found to be more stable. Harsh environment, i.e. multiple freeze-thaw cycles, strong acid, base and RNase treatment had hardly any effect on the levels of lncRNAs [16]. Thus, lncRNAs can be readily detected by qPCR from the body fluids, i.e. serum, plasma, gastric liquids, or urine [17]. But blood and gastric liquid collection is invasive. Urine collection is not applicable for anuretic patients. Saliva collection is non-invasive, simple, low-cost, practical and comfortable compared with other invasive methods. Thus, saliva is an ideal body fluid for biomarker detection in clinical applications. It is unclear whether the cancer-specific RNAs detected in the circulation originate from tumour cell death and lysis or whether they are actively secreted by tumour cells or blood cells. Although dozens of molecular biomarkers in saliva were identified for detecting different diseases, the rationale and relationship between systemic diseases and saliva biomarkers are unclear. Gao et al. [18] developed a panel of salivary transcriptomic biomarkers discriminatory for melanoma and lung cancer in the tumour-bearing rodent model. They speculate that systemic networks are present in our body, which allows communication between distal organs and the salivary glands. Signals transmitted through such networks can activate related signaling pathways that lead to altered gene expression and thereby produce disease-induced salivary biomarker profiles. Such disease-induced salivary gland-mediated transcriptomes can serve as valuable biomarker of disease onset and/or progression. Therefore, the salivary glands can be regarded as a reactive organ monitoring systemic diseases and saliva can be investigated as a biomarker-enriched disease-reflective biofluid. Zhang et al. [19] hypothesize that tumours produce mediators (hormones/cytokines/lymphokines) which can regulate the activities and gene expression patterns of distal organs (salivary glands) through the circulation. Upon contact with the salivary gland, cancer-specific mediators elicit altered gene expression profiles and transcription of associated RNAs that are secreted into the saliva. Both HOTAIR and PVT1 were elevated in PC tissue and saliva of PC patients. So they may be mainly derived from PC cells. HOTTIP was increased in PC tissue but not in saliva of PC patients in this study. According to aforementioned theories, we reason that through the circulation, PC-produced mediators contact the salivary glands, and subsequently elicit altered expression of HOTAIR and PVT1 genes but not HOTTIP gene.

Limitations exist in this study. First, microarray was not conducted to make lncRNA profiles of PC. So other potential lncRNA biomarkers may also exist. Second the sample size was small, and the subjects were limited to a Chinese Han population. Multi-centre studies with larger sample sizes are needed to further validate our findings. Third, the biological functions of HOTAIR and PVT1 were based on previous reports. In-depth analysis of their biological functions is needed. Fourth, saliva samples of patients with recurrent PC after the pancreatectomy were not collected. Thus, salivary levels of HOTAIR and PVT1 after PC recurrence need further investigation in the future.

In conclusion, differential expression of salivary HOTAIR and PVT1 may closely correlate with distal pancreatic cancer, and they can be novel non-invasive biomarkers with good sensitivity and specificity, better than serum CA19-9 for detecting PC. This study provides a new method and prospect for the early detection of pancreatic cancer or other systemic diseases using a non-invasive screening method.

## MATERIALS AND METHODS

### Subject selection

From 2011 to 2015, 18 tissue samples consisted of six samples of PC tissue, six tissues of benign pancreatic tumour (BPT), and six normal pancreatic tissues (NPT) resected from adjacent BPT. Saliva was collected as previous described [20, 21]. Saliva samples from 55 pre-operative patients with resectable PC, and 20 with BPT were collected. Six saliva samples from post-operative PC patients over six months after the curative panreatectomy were also procured. At time of the post-operative sample procurement, no cancer recurrence was implicated in the CT or MRI examinations of the six patients. Total 48 saliva samples of eight leading cancer types worldwide (six samples from each cancer) [14]: lung, breast, liver, stomach, colon, esophagus, prostate, and cervix uteri cancers (six samples from each group) were also obtained. All patient histopathology results were confirmed by pathology, and no concurrent oral and other systemic diseases were diagnosed, such as gingivitis, tuberculosis, systemic lupus erythematosus et al. Patients with a diagnosis of other malignancies and receiving chemotherapy and radiotherapy prior to saliva collection were not recruited neither. Pancreatic cancers were either resectable or borderline resectable according to National Comprehensive Cancer Network: Practice Guidelines in Oncology v.2.2010 [22]. Details are presented in Supplementary Material 1. Cancer staging was based on the American Joint Committee on Cancer: the 7th Edition of the AJCC Cancer Staging [23]. Fifty-five saliva samples from healthy individuals matched with PC patients in age, gender, and ethnicity were obtained. Institutional review boards or ethics committees at all participating institutes approved the study protocol. All participants were provided written consent for their information to be stored in the hospital database. Brief demographic information of all patients is presented in Supplementary Material 2.

### Protocols for the detection of lncRNAs

Total RNAs were extracted from frozen tissues and 1.2 ml of saliva using TRIzol or the mirVana PARIS Kit, respectively (both from Life Technologies, USA) according to the manufacturer's protocols. The reverse transcription reaction of lncRNAs was performed with the ReverTra Ace qPCR RT Kit (Toyobo, Japan) and the cDNA solution was amplified using Maxima SYBR Green qPCR master mixes (Thermo Fisher Scientific, USA) following the manufacturer's protocols. qPCR reactions were run using an ABI 7500 Real-Time PCR System (Life Technologies, USA), and the reaction mixtures were incubated at 95°C for 10 min, followed by 45 cycles of 95°C or 15 s and 60°C for 32 s. Melt curve analysis was performed to validate the generation of the expected PCR products. Each sample was analyzed in triplicate. The expression levels of each lncRNA were normalized to that of β-actin. All expression levels were calculated using the 2^−ΔΔCt^ method.

### Study design

This study consisted of a discovery phase and a verification phase, followed by a validation phase. Five well-documented lncRNAs (H19, HOTAIR, HOTTIP, MALAT1, PVT1) most closely associated with PC from previous studies were selected as putative lncRNA biomarkers. In the discovery phase, the expression levels of the selected lncRNAs were measured by qPCR and compared within PCT, BPT tissues and NPT.

In the verification phase, the five lncRNAs were also subjected to validation by qPCR using a saliva sample set containing 55 patients with PC, 20 with BPT and 55 healthy controls. HOTAIR and PVT1 exhibiting statistically significance when comparing PC group with healthy or BPT group were identified as potential salivary biomarkers to detect PC.

In the validation phase, in order to preliminarily evaluate the specificities of the two potential salivary biomarkers, their expression levels were assessed in total 48 saliva samples of eight leading causes of cancer-related deaths worldwide, including lung, breast, liver, stomach, colon, esophagus, prostate, cervix uteri cancers, and compared with that of healthy controls.

### Statistical analysis

LncRNA expression levels and ages were compared using the Mann Whitney *U* test. Genders were compared using the χ^2^ test. The differences of lncRNAs before and after surgery were studied by Wilcoxon signed-rank test. ROC curves were used to evaluate the discriminatory power of each lncRNA for differentiating between two groups. The validated salivary biomarkers were constructed into binary logistic regression models, and step-wise backward model selection was performed to determine the final combinations of biomarkers. Statistical analyses were performed using the SPSS software (ver. 13.0). A two-tailed *P* value < 0.05 was considered statistically significant.

### SUPPLEMENTARY FIGURES AND TABLES




