# Supplementary material for: Salivary HOTAIR and PVT1 as novel biomarkers for early pancreatic cancer
Source: Oncotarget. 2016 Mar 23;7(18):25408–19. doi: 10.18632/oncotarget.8323 (PMC5041913; doi:10.18632/oncotarget.8323)
Supplement: Supplementary file 1 [file oncotarget-07-25408-s001.pdf]

Salivary HOTAIR and PVT1 as novel biomarkers for early pancreatic cancer

Supplementary Material

CRITERIA DEFINING RESECTABILITY STATUS

Tumors considered localized and resectable should demonstrate the following:

- No distant metastases
- No radiographic evidence of superior mesenteric vein (SMV) and portal vein abutment, distortion, tumor thrombus, or venous encasement
- Clear fat planes around the celiac axis, hepatic artery, and SMA.

Tumors considered borderline resectable include the following:

- No distant metastases
- Venous involvement of the SMV/portal vein demonstrating tumor abutment with or without impingement and narrowing of the lumen, encasement of the SMV/portal vein but without encasement of the nearby arteries, or short segment venous occlusion resulting from either tumor thrombus or encasement but with suitable vessel proximal and distal to the area of vessel involvement, allowing for safe resection and reconstruction.
- Gastroduodenal artery encasement up to the hepatic artery with either short segment encasement or direct abutment of the hepatic artery, without extension to the celiac axis.
- Tumor abutment of the SMA not to exceed greater than 180 degrees of the circumference of the vessel wall.

NCCN Pancreatic Adenocarcinoma Panel recognize the work of the experts and adapt their criteria to define resectability status. Callery MP, Chang KJ, Fishman EK, et al. Pretreatment Assessment of Resectable and Borderline Resectable Pancreatic Cancer: Expert Consensus Statement. Ann Surg Oncol 2009;16:1727-1733.

Tumors considered to be unresectable demonstrate the following:

- HEAD
  - Distant metastases
  - Greater than 180 degrees SMA encasement, any celiac abutment
  - Unreconstructible SMV/portal occlusion
  - Aortic invasion or encasement
- BODY
  - Distant metastases
  - SMA or celiac encasement greater than 180 degrees
  - Unreconstructible SMV/portal occlusion
  - Aortic invasion
- TAIL
  - Distant metastases
  - SMA or celiac encasement greater than 180 degrees
- Nodal status
  - Metastases to lymph nodes beyond the field of resection should be considered unresectable.

Note: All recommendations are category 2A unless otherwise indicated.
Clinical Trials: NCCN believes that the best management of any cancer patient is in a clinical trial. Participation in clinical trials is especially encouraged.

Supplementary Material S1: The criteria defining the resectability status of panceatic cancer according to the NCCN: Practice Guidelines in Oncology v.2.2010.

Supplementary Material S2: The demographic and clinical information of the enrolled patients.
